# The Protein Toxins Ricin and Shiga Toxin as Tools to Explore Cellular Mechanisms of Internalization and Intracellular Transport

**DOI:** 10.3390/toxins13060377

**Published:** 2021-05-25

**Authors:** Kirsten Sandvig, Simona Kavaliauskiene, Tore Skotland

**Affiliations:** 1 Department of Molecular Cell Biology, Institute for Cancer Research, Oslo University Hospital, The Norwegian Radium Hospital, 0379 Oslo, Norway; simona.kavaliauskiene@medisin.uio.no (S.K.); toresko@uio.no (T.S.); 2 Department of Biosciences, University of Oslo, 0315 Oslo, Norway

**Keywords:** endocytosis, intracellular transport, Golgi apparatus, endoplasmic reticulum, membranes, lipids, mass spectrometry

## Abstract

Protein toxins secreted by bacteria and found in plants can be threats to human health. However, their extreme toxicity can also be exploited in different ways, e.g., to produce hybrid toxins directed against cancer cells and to study transport mechanisms in cells. Investigations during the last decades have shown how powerful these molecules are as tools in cell biological research. Here, we first present a partly historical overview, with emphasis on Shiga toxin and ricin, of how such toxins have been used to characterize processes and proteins of importance for their trafficking. In the second half of the article, we describe how one can now use toxins to investigate the role of lipid classes for intracellular transport. In recent years, it has become possible to quantify hundreds of lipid species using mass spectrometry analysis. Thus, it is also now possible to explore the importance of lipid species in intracellular transport. The detailed analyses of changes in lipids seen under conditions of inhibited toxin transport reveal previously unknown connections between syntheses of lipid classes and demonstrate the ability of cells to compensate under given conditions.

## 1. Introduction

**Figure 1:**
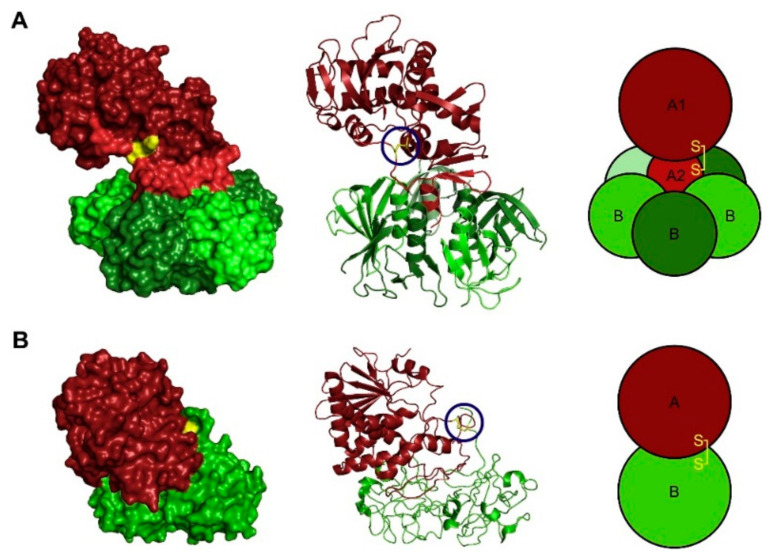
The structure of (**A**) Shiga toxin (PDB ID:1DM0 [4] and (**B**) ricin (PDB ID: 2AAI) [5] determined by X-ray crystallography. The enzymatically active A moieties are colored red, and the B moieties are colored green. The A1 fragment of Shiga toxin is a darker red than the A2 fragment. The disulfide bridge linking the enzymatically active part to the rest of the toxin is indicated in yellow and marked with blue circles in the ribbon structure. Reprinted with permission from ref. [6] Copyright 2014 Elsevier.

A number of protein toxins from plants or secreted by bacteria efficiently intoxicate cells by binding to the cell surface and, after entry into the cytosol they inhibit the protein synthesis enzymatically, thereby killing the cell. Several of these toxins consist of two moieties, one that binds to cells and another that has enzymatic activity (for review, see [1]). Examples of two such toxins, the plant toxin ricin, which is found in the seeds of *Ricinus communis*, and the bacterial toxin Shiga toxin, which is secreted by *Shigella dysenteriae*, are shown in Figure 1. The enzymatically active moiety can, in both cases, inactivate the 60S subunit of the ribosome by removal of one adenine from the 28S RNA [2,3]. Interestingly, several other plant toxins have a similar two-chain structure connected by a disulfide bridge. Although these toxins come from different plants, they share the ability to inactivate ribosomes in a similar manner. Such toxins are abrin, modeccin, viscumin, and volkensin [1].

**Figure 2:**
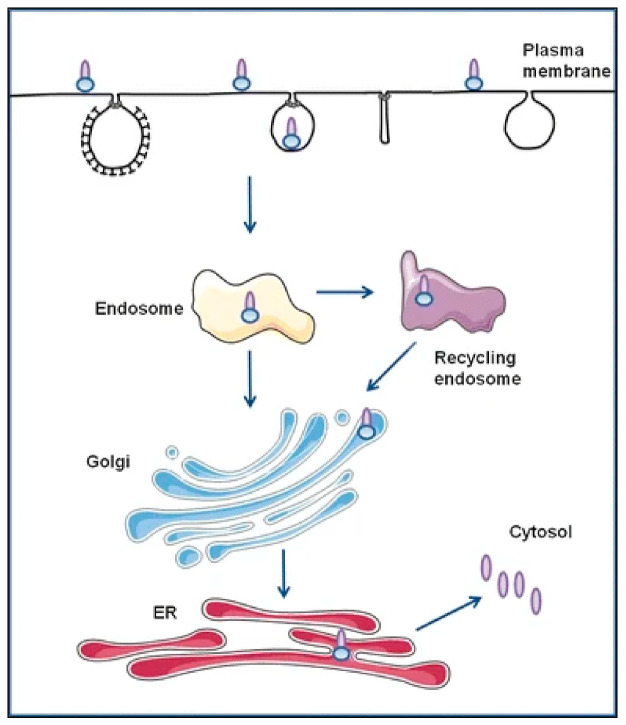
Endocytosis and retrograde transport of Shiga toxin and ricin. Both toxins bind to cell surface receptors. Shiga toxin binds to the glycosphingolipid Gb3, and ricin binds to the terminal galactose of glycolipids or glycoproteins. After being endocytosed, the toxins are transported directly to the Golgi apparatus or via the recycling endosomes before they are further transported to the ER, where the A-moiety (A1-fragment for Shiga toxin) is released and translocated to the cytosol. Once in the cytosol, the active A-chain inhibits protein synthesis by removing one adenine from the 28S RNA of the 60S subunit of the ribosome. Note that recycling and transport to lysosomes are not shown. Reprinted with permission from ref. [16] Copyright 2013 Springer.

About 40 years ago, researchers started to obtain information about how these toxins were able to become translocated to the cytosol. Data obtained at that time showed that some toxins appeared to enter the cytosol after endocytic uptake. For diphtheria toxin, another bacterial toxin able to inhibit protein synthesis (although by ADP-ribosylation of elongation factor 2) [7], there was some evidence for transfer into the cytosol after uptake since ammonium chloride, known to increase lysosomal pH, protected cells against intoxication [8]. In 1980, it was published that diphtheria toxin could enter directly through the cell membrane when cells with surface-bound nicked diphtheria toxin were exposed to low pH [9,10]. This finding paved the road for similar investigations of several other toxins that also undergo a low pH-induced conformational change and subsequent translocation through the membrane [1]. What about other toxins that do not require low endosomal pH for entry into the cytosol? Is endocytosis irrelevant for the cytosolic entry? The structure of cholera toxin with a ring of B-subunits and the A-moiety in the middle led scientists to suggest that the A-moiety could somehow sink through the membrane and enter the cytosol. However, as we know today, this is not the case. In 1982, it was shown that ricin endocytosed under protective conditions, such as Ca^2+^ deprivation or pH 6.0, intoxicated cells when the protection was released [11]. This demonstrated that endocytosed toxin was able to enter the cytosol, and later studies revealed that several toxins, including Shiga toxin and ricin, are transferred to the Golgi and the ER before translocation to the cytosol (Figure 2). Actually, Shiga toxin was the first toxin found to be transported all the way from the cell surface to the Golgi and the ER [12]. The ability of these protein toxins to be translocated from the cell surface and retrogradely to the ER from where the A moieties are released have made them excellent tools to study the processes and transport steps involved. Their final effect on protein synthesis provides us with a very sensitive test system for the arrival in the cytosol. Thus, searching for compounds that protect against protein toxins not only can be of benefit in connection with disease or intoxication by these toxins but can help us to clarify transport pathways in the cells. The construction of genetically modified toxin molecules with sulfation sites, which are modified in the trans-Golgi, and glycosylation sites that can be modified in the ER [13,14] has been of great help in revealing which step is affected. Thus, toxins have contributed to our knowledge about transport from the cell surface to endosomes, to the Golgi apparatus, and to the ER and nuclear envelope. The toxins can also be exploited to investigate how cells are affected by exposure to other treatments. For instance, they have been used to show that nanoparticle uptake may change other cellular pathways [15].

Importantly, protein toxins can be exploited in medicine [17,18,19,20]. Researchers are attempting to use protein toxins for the selective killing of cancer cells by making constructs containing at least the enzymatically active part of the toxin and either an antibody against epitopes on cancer cells or growth factors for which the corresponding receptors are expressed at a high level on the target cells. So far, some products containing the active part of diphtheria toxin and *Pseudomonas* exotoxin A have been approved for human use [19,21]. The neutral glycosphingolipid Gb3, which serves as the receptor for Shiga toxin, is mostly found on cancer cells [17,18]. Therefore, this toxin can be used to detect cancer and to target drugs to cancer cells. One study taking advantage of toxin transport to the ER for release of a cancerostatic drug was published some years ago [22], and it will be interesting to see further development in the field of toxin targeting.

**Figure 3:**
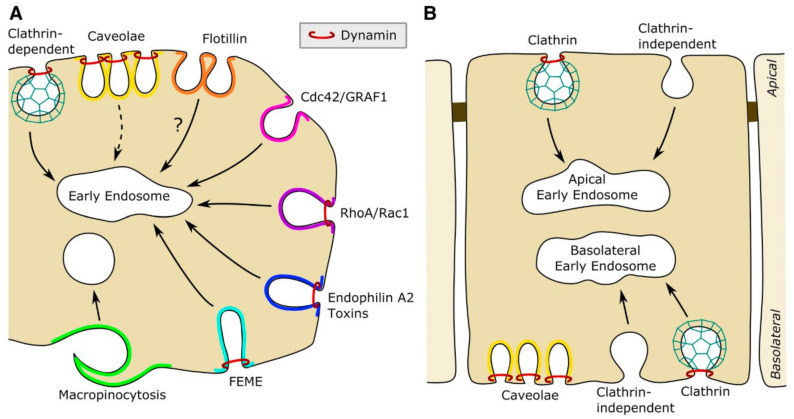
An overview of endocytic mechanisms in a non-polarized cell (**A**) and a polarized cell (**B**). We have indicated some pathways such as clathrin-dependent endocytosis, caveolae (now regarded to be quite stable structures), Cdc42/GRAF1, and others. It should be noted that clathrin-independent uptake is regulated in different ways on the apical and basolateral side as described in the text. It should be noted that in MDCK cells all caveolae are on the basolateral side. Reprinted from an open-access review [27].

As described below, our current view on the complexity of endocytic mechanisms, as illustrated in Figure 3, has been developed partly by studies of protein toxins such as ricin and Shiga toxin. Concerning the mechanisms seen in Figure 3, we refer to the following new articles for further details: Clathrin-dependent endocytosis [23,24]; the Cdc42- dependent pathway, which can account for 70% of fluid uptake [25]; an updated review concerning FEME (Fast Endophilin-Mediated Endocytosis) [26]; and, for a more general review, [27].

## 2. Cellular Pathways Exploited by Toxins

This section describes some historical aspects of the scientific progress in cell biology involving toxins, with emphasis on ricin and Shiga toxin. The description also provides a background for how the toxins can be used to clarify the importance of membrane lipids for intracellular transport and cell physiology, which is discussed in Section 3.

### 2.1. Endocytic Mechanisms and Recycling

The protein toxin ricin binds to a variety of molecules with terminal galactose. As described in the current review, the toxin has provided us with important information about several cellular mechanisms. More than 40 years ago, we found that ricin could easily be labeled with radioactive iodine by an enzymatic method (lactoperoxidase), which preserved its binding affinity to the cell surface, as well as its toxicity [28]. This contrasted with labeling using strong oxidizing conditions, which reduced the activity of ricin. Furthermore, ricin can easily be removed from the cell surface with addition of lactose, thereby facilitating its use in studies of endocytosis. Early studies revealed that there was not any strict temperature cut-off for uptake of ricin [29] and, furthermore, that endocytosis of fluid phase and ricin is dependent on cell density [30,31,32]. Cell density was later shown to affect the lipid composition of cells [33,34]. Furthermore, these early studies revealed that endocytic uptake is not a one-way street, as ricin that was internalized could come out of the cell again in an intact form [29,35]. The same was the case for ricin B-chain, showing that the recycling was unrelated to the toxicity of ricin. Since these initial studies of ricin recycling, knowledge about different recycling mechanisms and the molecules involved has exploded, and a review about the complexity of these processes was recently published by Weeratunga et al. [36].

Clathrin-independent uptake of a toxin was first suggested for cholera toxin, which could be observed in caveolae by EM [37], and subsequently inside cells in endocytic vesicles. It was suggested that these organelles, which have a characteristic appearance as flask-shaped invaginations with a diameter of 60–80 nm [38], were responsible for uptake of the toxin. Since cholera toxin can induce a transmembrane Ca^2+^-flux and kinase activation [39,40,41] it possible that the toxin in itself may affect the pinching off of caveolae. Also, at that time, it was not excluded that the toxin was stuck in this location and, at least to some extent, rapidly taken up by other mechanisms such as clathrin-dependent endocytosis. Later studies revealed that cholera toxin, in fact, can be internalized by different mechanisms, and both clathrin-dependent and clathrin- and dynamin-independent uptake mechanisms can be involved [42,43,44]. Uptake from caveolae is normally not a very efficient process [45]. However, caveolae can pinch off, and the content is then transferred to early endosomes. It was initially published that the content from caveolae ended up in neutral structures called “caveosomes,” and from there, the content was transported to the endoplasmic reticulum [46]. However, the caveosomes were later found to be artefacts [47,48], and the authors suggested that the term should no longer be used. In spite of this, researchers continue to describe that they may target, for instance, nanoparticles to “caveosomes” to avoid lysosomal degradation [49]. Although a virus such as SV40 may cause signaling and entry through the caveolae [50], this virus can also enter cells by other endocytic mechanisms [51]. Interestingly, it has been demonstrated that caveolin can stabilize certain ligands at the cell surface. This is the case, for instance, for autocrine mobility factor (AMF) and cholera toxin [52]. It should be noted that a new role for caveolae has recently been characterized: Caveolae can provide additional membrane upon mechanical stress and reform in an ATP-dependent manner [38,53].

During the 1980s, ricin played an important role in showing evidence for the existence of clathrin-independent endocytosis. When cells are exposed to a hypotonic shock and potassium-depletion, some cell types lose the clathrin-coat at the cell membrane [54]. After exposure of cells to such a treatment, ricin could still end up in an intracellular compartment unavailable to antibodies, and subsequently intoxicate cells [55]. Similarly, when the cytosol was acidified—a condition that blocks pinching off of clathrin-coated pits—ricin was endocytosed in several different cell types [56]. However, early estimates suggested that clathrin-coated pits could account for all uptake into the cells [57]. At that time, a number of arguments were used to raise doubts about the existence of clathrin-independent uptake. For instance, when clathrin is released from the membrane after potassium depletion, is there still a sufficient uptake mechanism to preserve endocytosis from the same structures? When you remove the clathrin coat or acidify the cytosol to block pinching off clathrin-coated pits, do you then induce alternative mechanisms? Even experiments with unperturbed cells, which showed that Concanavalin A and transferrin ended up in separate vesicles soon after uptake, did not seem to help [58]. It was not until the Schmid-lab showed that fluid phase uptake continued even when clathrin-dependent uptake was blocked by the expression of a dominant negative mutant of dynamin [59,60] that the resistance against existence of clathrin-independent endocytosis seemed to disappear. Thus, a change in opinion in the field can take several years. The ability of cells to regulate endocytic pathways in response to changes in other uptake mechanisms is a challenge when studying such mechanisms. Fluid uptake continues at a similar rate after a block in clathrin-dependent endocytosis due to compensatory mechanisms [59,60], and later studies have revealed a co-regulation of caveolar and Cdc42-dependent fluid phase endocytosis by phosphocaveolin-1 [61]. Upon reduction of phosphocaveolin-1, there is an increase in the Cdc42 dependent uptake and vice versa. Cavin has also been reported to downregulate the Cdc42-dependent pathway [62]. Certainly, later studies have demonstrated the complexity of uptake mechanisms [26,27,63].

It should be noted that clathrin-independent endocytosis in polarized cells is subjected to a differential regulation on the apical versus basolateral side. When ricin was used to study uptake from the two poles in polarized MDCK cells, the apical endocytosis was upregulated by protein kinase A, protein kinase C, cyclooxygenase, and the inhibition of calmodulin. Under the same conditions, there was no effect on the basolateral uptake (for review, see [27]).

Caveolae are known to be dependent on cholesterol, and removal of this lipid or addition of filipin, which binds to cholesterol, rapidly destroys these structures [64,65]. However, experiments with ricin and addition of methyl-β-cyclodextrin, which extracts cholesterol from the cell membrane, showed that also macropinocytosis is sensitive to a reduction of this lipid [66]. Furthermore, invagination of clathrin-coated pits is inhibited by the addition of methyl-β-cyclodextrin [67,68]. Even under such conditions, ricin can still be endocytosed by other mechanisms (for review, see [27]). In addition, the literature shows that the inhibition of uptake of a certain ligand by reduction of cholesterol is taken as a proof for uptake of that ligand by caveolae. When reporting that the uptake of rather large nanoparticles (far larger than the diameter of caveolae) occurs by this mechanism due to a reduction of the uptake after addition of methyl-β-cyclodextrin, caution should be exercised before drawing any conclusions.

Shiga toxin, which binds to the neutral glycosphingolipid Gb3, has taught us several lessons in cell biology. The toxin can be endocytosed by different endocytic mechanisms, and it can clearly affect its own uptake (for reviews, see [69,70,71,72]). It was the first toxin shown to be able to aggregate in clathrin-coated pits [73] in spite of binding to a lipid that does not seem to interact with surface proteins. Later studies applying different methods to interfere with clathrin-dependent endocytosis, including the expression of a dominant negative mutant of dynamin and inducible expression of antisense RNA to clathrin heavy chain, have supported this finding [74,75]. Furthermore, the expression of both dominant negative mutants of epsin and eps15 reduced Shiga toxin uptake [43]. The mechanism behind the toxin aggregation in clathrin-coated pits is still unknown. Although Gb3 does not transverse the membrane, the toxin is able to induce activation of kinases [76,77,78] and phosphorylation of clathrin [76]. Shiga toxin is also able to increase the number of clathrin-coated pits, and it can affect the lifetime of these structures [79]. Furthermore, it should be noted that Shiga toxin uptake is increased at high toxin concentrations in several cell lines. This increase is dependent on the A-subunit and seems to occur through clathrin-coated pits, as it did not occur in BHK cells after induction of antisense to clathrin heavy chain [80]. Although Shiga toxin can be endocytosed from clathrin-coated pits, the fraction using this uptake mechanism is likely to be cell-type dependent. Upon reduction of accumulation of Shiga toxin in clathrin-coated pits, the toxin may more easily be taken up by other mechanisms. In addition, since interfering with clathrin-dependent uptake also may upregulate other mechanisms, the significance of clathrin-dependent uptake is likely to be underestimated. There are still several questions to answer when it comes to uptake of Shiga toxin from clathrin-coated pits: Does this occur mainly from coats with or without AP2 [81]? Which other proteins or protein-modifications associated with clathrin-coated pits could be required for this process? Is the interaction between the outer membrane leaflet, which contains Gb3, and the internal membrane lipids such as phosphatidylserine (PS) or phosphatidylinositolphosphates (PIPs) required? Are specific lipid species important for this process? We recently published a review describing how certain species of PS can play a role for interaction with cytosolic proteins [82]. Recently, PS was found to be important for the formation of clathrin-coated vesicles [83]. The complex formation and disappearance of PIPs during formation of clathrin-coated vesicles might similarly be involved [23]. Thus, further studies using Shiga toxin in this connection are likely to provide more information on cell biology.

Shiga toxin was many years ago found to be able to induce invaginations at the cell surface [84], and it was shown to be taken up from such structures in a dynamin-, cholesterol-, and energy-dependent manner. Later on, it was shown that the process is dependent on endophilin A2 and actin and somewhat related to the endocytic pathway called FEME [26], although the latter is dependent on growth factor activation. Shiga toxin uptake has, on the other hand, been found to be dependent on toxin-induced curvature changes, a process studied by Johannes and coworkers within recent years [69].

### 2.2. Endosome to Golgi Transport

Toxins have turned out to be extremely useful to study endosome to Golgi transport. Several toxins need to be translocated to the Golgi on their way to the cytosol, and a useful early tool indicating the importance of this transport step for intoxication was the drug Brefeldin A (BFA), which, in many cell types, disrupts most of the Golgi apparatus and induces transport of Golgi cisternae back to the ER [85,86,87]. However, not all cells have a BFA-sensitive Golgi apparatus [88,89,90], and the drug BFA selectively protects against intoxication in cells with a BFA-sensitive Golgi apparatus [90]. Thus, one can study the effects of different molecules or drugs on cell intoxication to learn about interference with this transport step. Importantly, chemical modifications of genetically modified ricin or Shiga B-chain with sulfate in the trans Golgi or by glycosylation of ricin in the ER [13,14] have facilitated our studies of this pathway. Moreover, electron and fluorescence microscopy have contributed to a large amount of the knowledge we have today about this transport. A combination of these methods is useful to validate the results, as molecules may be observed in a structure without necessarily moving through that structure. On the other hand, molecules can be transferred by short-lived efficient carriers, which are difficult to observe. Also, if the treatment of the cells changes the level of sulfation or glycosylation, then there is no easy way to “compensate” for this change, which could be due to reduced transport of cellular molecules through the cell and therefore less competition for sulfation or glycosylation. Alternatively, it could be due to lower activity of the enzymes involved.

Studies performed many years ago with ricin and Shiga toxin (or the Shiga B chain) have revealed that, on their way from endosomes to the Golgi apparatus, toxin molecules did not have to be transported via late endosomes [91,92]. It is interesting to see in the literature, that Shiga B chain is now mentioned as a well-established tool to study transport to the Golgi apparatus. An important difference between ricin and Shiga toxin is that the endosomal protein GPP130 was identified as a Shiga toxin-binding protein that helps in bringing this toxin to the Golgi apparatus [93,94]. It should be noted that GPP130 does not interact with Shiga-like toxin 2 (Stx2), which is important for disease caused by toxin-secreting *E. coli* species, and the mechanism behind transport of this toxin to the Golgi is not known. Over the years, different kinases, sorting nexins, Rab-proteins, Golgins, and SNARE-complexes have been shown to be involved in endosome to Golgi transport (for review, see [95,96]). For instance, in the case of Shiga toxin, Rab6A’ was found to be required for endosome to Golgi transport [97,98]. For ricin, both Rab6A and Rab6A’ seem to be involved in this transport step, as the best inhibition of transport was obtained by knocking down both isoforms [99]. Importantly, these studies have also illustrated the ability of cells to compensate. When knockdown of Rab6A was between 40% and 75%, an inhibition of transport was observed, but if knockdown was better than 75%, then there was an upregulation of Rab6A’ and no inhibition of transport. Early experiments with PI-3 kinase inhibitors suggested an involvement of this enzyme for ricin transport to the Golgi [100], and this was later confirmed in studies with hVps34 mutants and siRNA [101]. At about the same time, different laboratories showed the involvement of sorting nexins for transport of both ricin and Shiga toxin to the Golgi apparatus [101,102,103], and the retromer and clathrin were also reported to be involved in Shiga toxin transport [104]. Shiga toxin also induces a dissociation of cPLA2α from a complex with Annexin A1, and the active free form of the enzyme can then facilitate Golgi transport [41]. The recycling compartment has been described to be an intermediate station for Shiga toxin on its way to the Golgi [105,106,107,108], but since this compartment seems structurally different in various cell types, its importance for toxin transport could vary.

### 2.3. Retrograde Toxin Transport from the Golgi to the ER and Translocation to the Cytosol

**Figure 4:**
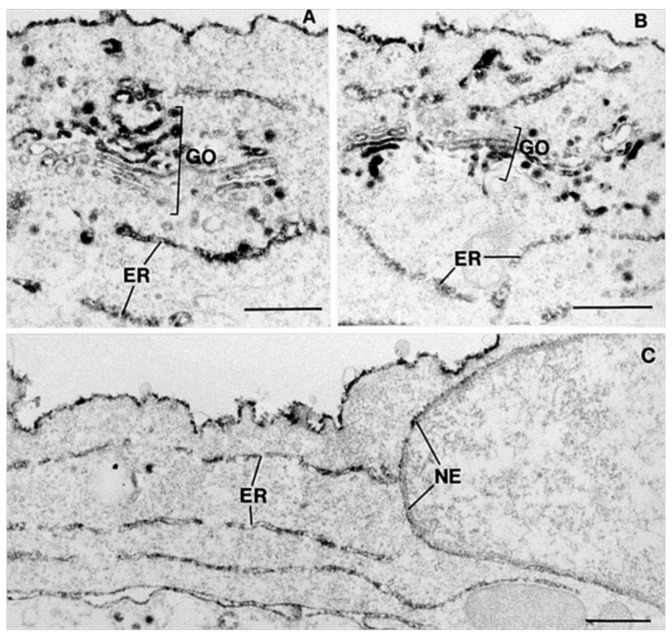
Intracellular localization of endocytosed Shiga toxin-HRP in A431 cells. Shiga toxin is observed in the Golgi cisternae (GO), the endoplasmic reticulum (ER) and the nuclear envelope (NE). Scale bars are 0.5 µm. Reproduced with permission from ref. [12] Copyright 1992 Springer Nature.

A well-studied mechanism for retrograde transport from the Golgi to the ER is the COPI-mediated transport of molecules containing a KDEL-sequence (for review, see [109,110]). However, neither Shiga toxin nor ricin have such a sequence, but are still able to go from the trans-Golgi network to the ER, as illustrated for Shiga toxin in Figure 4. Studies with retrograde transport of Shiga toxin have elucidated the complexity of retrograde transport in general, since Shiga toxin was found to be transported to the ER by a COPI-independent, Rab6-dependent pathway [111,112,113]. Later studies of Shiga toxin and ricin have used siRNA or shRNA to add other candidates to the list of molecules that may be involved in retrograde transport [114,115]. This includes COPII, TRAPP, and GARP complexes. However, it is important to be aware of that some of the effects could be indirect. For example, an incorrect sorting of the enzyme furin, involved in cleavage and activation of Shiga toxin [116], might lead to protection but may not necessarily affect toxin transport (for a discussion of these studies, see [16]). Furthermore, changes of the Golgi apparatus, for instance, disruption of this organelle due to expression of a temperature-sensitive ∈-COP subunit, were found to induce an alternative BFA-resistant transport pathway for ricin to the ER, illustrating the ability of the cell to compensate for blocks in transport [117].

Several different molecules have now been identified to be involved in retrograde transport and for translocation of the enzymatically active part of the toxins (for review, see [95,96]). Again, it is important to be aware of the ability of cells to induce compensatory processes. For instance, Sec61 was not found in a screening for molecules able to protect against ricin [115] but was previously found to be able to interact with ricin [118,119,120]. Consequently, one may wonder if an alternative translocation mechanism is somehow operating under those conditions. However, the screening indicated the importance of derlins, and it is possible that several molecules can support translocation to the cytosol. This is in contrast to Shiga toxin, which has also been reported to be in a complex with Sec61 [121]. Screening studies have also supported the idea that Sec61 is involved in transport to the cytosol [115].

The compound Retro-2 is an example of how identification of compounds that protect against ricin and Shiga toxin may provide us with basic knowledge about general mechanisms. This drug provides very good protection against Shiga toxin, apparently due to lack of transport of GPP130 from endosomes to the Golgi apparatus [122]. This is associated with its ability to target the ER component Sec16A and prevent transport of syntaxin 5 from ER to the Golgi [122]. However, the drug also protects against ricin [123], but the explanation for this protection is not obvious. Future studies are likely to provide more information about the relation of syntaxin 5 to other SNARE complexes and transport in the ER-Golgi area. Interestingly, Retro-2 was reported to inhibit ASNA-1 mediated ER targeting and membrane insertion of tail-anchored proteins [124], a finding that may be related to protection against ricin. Ricin transport from endosomes to the Golgi apparatus was reported to be inhibited only by 20% upon knockdown of ASNA-1 [125]. Thus, ricin transport seems to be blocked at a later step in the retrograde pathway than Shiga toxin. In 1995, it was reported that transient expression of mutants of GTPases Sar1, ARF1, and Rab1, protected against ricin, thereby interfering with ER-Golgi trafficking [126]. Whether signaling alterations in the ER induced by changes in the ER exit sites [127] are related to the protection against ricin remains to be elucidated.

Shiga toxins produced by bacteria are still a threat to human health, as is intoxication with ricin. Thus, the clarification of how the toxins work, as well as a search for compounds that protect against them, are important. In a review by Kavaliauskiene et al. [72], chloroquine and hydroxychloroquine, both drugs used in humans, were able to protect against both Shiga toxin and Stx2. In a recent review by Selyunin and Mykhopadhyay [95], several other drugs, which are all approved for human use, were also described. However, it is not clear whether all these drugs act in a similar manner. Chloroquine might inhibit translocation from the ER, whereas drugs such as tamoxifen seem to inhibit transport to the Golgi. In Section 3.7, we describe how several other compounds, such as glucose analogues, lysolipids, and a precursor of ether lipids, protect cells against both Shiga toxin and Stx2 by exerting changes in trafficking. It will be interesting to see whether such drugs might come into use in connection with infectious disease.

## 3. Role of Lipids for Membrane Function and Intracellular Transport

Here, we provide a short background about lipids and their importance for membrane structure, including the importance of the lipid composition of cellular membranes for the transport and toxicity of Shiga toxin and ricin. We then describe how studies performed by interfering with lipid metabolism have contributed to our understanding of membrane transport and revealed new aspects of lipid metabolism.

### 3.1. Lipid Classes and Species

**Figure 5:**
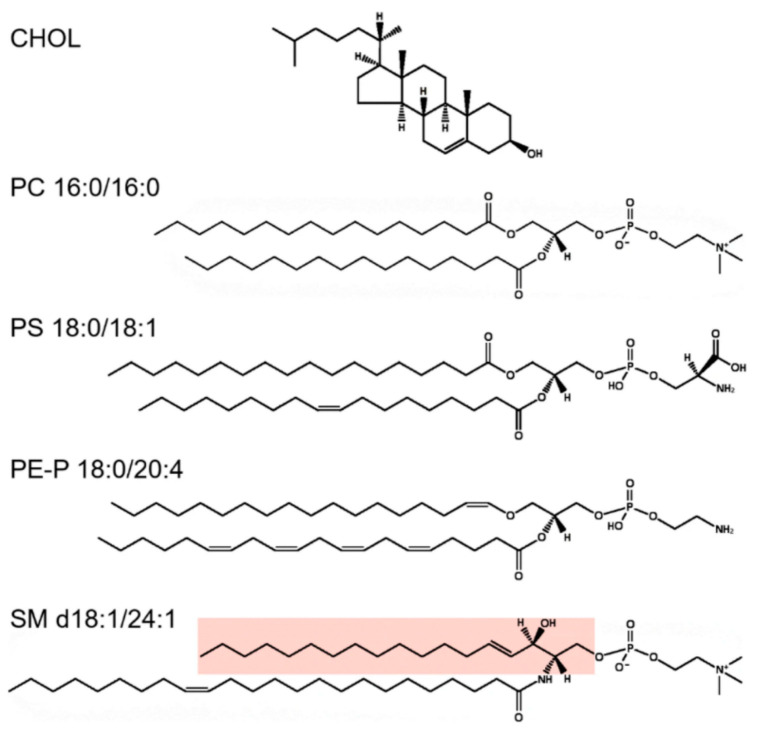
Illustrations of some lipid structures. On the top, cholesterol (CHOL) is shown, followed by PC 16:0/16:0, which is a lipid species often used in model membranes. However, although PC species are very common in cells, PLs normally contain very little of species with two saturated fatty acyl groups. PS 18:0/18:1 is an example of a PL with 1 saturated and 1 unsaturated fatty acyl group, which is the most common combination of fatty acyl groups in all PL classes. Note that all double bonds in PLs are in a *cis*-configuration and that the unsaturated fatty acyl group is most often found in the *sn-2* position. PE-P 18:0/20:4 is an example of a plasmalogen, i.e., an ether lipid with an alkenyl group, and these lipids often contain polyunsaturated fatty acyl groups in the *sn-2* position. The sphingolipid SM d18:1/24:1 is shown with the sphingosine part marked in pink. Note that the N-amidated fatty acyl group is so long that it can theoretically penetrate approximately halfway into the opposite leaflet. These structures were made using the drawing tool available at Lipid Maps (https://www.lipidmaps.org/ (accessed on 24 May 2021)). Reprinted from the open-access review [132].

Membrane lipids are grouped into different classes based on different head groups, such as the main phospholipid (PL) classes: Phosphatidylcholine (PC), phosphatidylserine (PS), phosphatidylethanolamine (PE), phosphatidylinositol (PI), and phosphatidic acid (PA). Each of the PL classes consists of many lipid species due to various compositions of fatty acyl groups, with different numbers of carbon atoms and double bonds (Figure 5). Thus, cells contain several thousands of different lipid species. With modern mass spectrometry (MS) analyses, it is possible to quantify several hundred or close to a thousand lipid species in one sample [128,129]. Most PLs contain ester-bound fatty acyl groups, but ether-linked PLs may constitute 5–20% of the lipids in some cells and are often referred to as “the forgotten lipids.” Most PLs contain C16 or C18 fatty acyl groups with zero, one, or two double bonds, although longer carbon chains with more double bonds (e.g., 20:4 and 22:6) are also common in some PL classes. For a recent and detailed review article describing the diversity of membrane lipids, see [130]. The biosynthesis of PLs and sphingolipids and where the different parts of these lipids are synthesized were reviewed and illustrated by the authors of [131].

**Figure 6:**
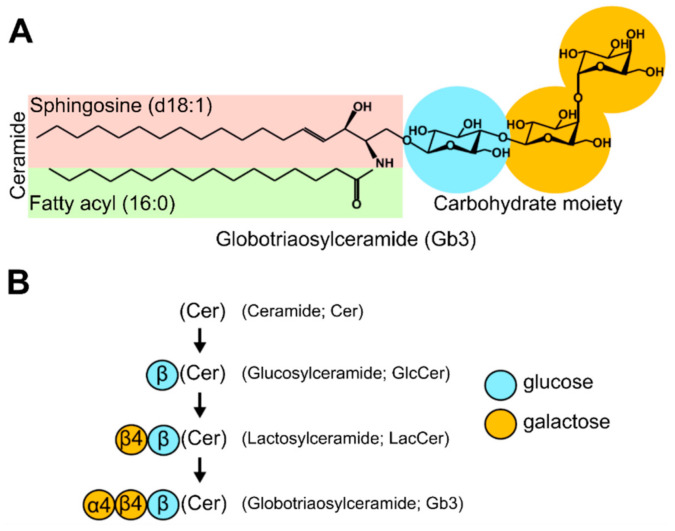
Structure of the Shiga receptor Gb3 (**A**) and synthesis of Gb3 from its precursors GlcCer and LacCer (**B**). The letter and number of the carbohydrate structure symbols describe the nature of the glycosidic linkage. Thus, β4 represents a β1-4 linkage to the carbohydrate on the right, and Gb3 is Galα1-4Galβ1-4GlcCer. Redrawn from [6] with approval from Elsevier.

Shiga toxin binds to the glycosphingolipid Gb3. Gb3 is synthesized from ceramide, and the structure of Gb3 and the precursors GlcCer and LacCer are shown in Figure 6. As shown in Figure 5, the sphingolipids differ from the PLs, as they have a sphingoid base backbone. Whereas the hydrophobic chains of the PLs are linked to a glycerol unit, the sphingolipids contain an N-amidated fatty acyl chain, where the N atom originates from serine. The fatty acyl groups of sphingolipids differ considerably from those in PLs and contain fatty acyl groups with 16 to 26 carbon atoms [133,134]. The most common glycosphingolipid species contain N-amidated C16:0, C24:0, and C24:1 (and some cells also have considerable amounts of C22:0) [33,82]. Although C16:0 is common also in PLs, C24:0 and C24:1 have not been reported to be present in PLs to our knowledge. Moreover, monounsaturated fatty acyl groups with 16–20 carbon atoms seem to be absent in glycosphingolipids [6,135]. However, minor amounts of Gb3 containing C18:1 were recently reported in H1299 cells [136]. Since the glycosphingolipids normally contain very little of species with N-amidated C18 and C20 fatty acyl groups, the glycosphingolipids have a bimodal distribution regarding the fatty acyl chain length [82], which is further discussed below.

Membrane lipids are amphiphilic with a hydrophilic head and hydrophobic tails. They form a bilayer membrane where the fatty acyl groups are in the middle, and the head groups are facing the surroundings on both sides. Importantly, the lipids are asymmetrically distributed in the cellular bilayers. For the present discussion, the asymmetric distribution of lipids in the plasma membrane (PM) is important. In the PM, probably all glycosphingolipids and most of SM and PC are found in the outer leaflet, whereas PS, PE, PI, and PA are almost exclusively located in the inner leaflet [137,138]. Although there may be considerable variations between the chain length and number of double bonds in the various PL classes, they most often contain a saturated fatty acyl group in the *sn-1* position and an unsaturated fatty acyl group in the sn-2 position.

Cholesterol is an important lipid in biological membranes, and may constitute 30–40 mol% of the lipids in the PM. Many researchers have studied interactions between cholesterol and other membrane lipids, as well as how cholesterol is distributed between the two leaflets. Some studies have suggested that almost all cholesterol is located mainly in either the inner or the outer leaflet of the PM, whereas other studies have reported that cholesterol is more or less equally distributed among the two leaflets. These diverting conclusions indicate that some of the methods used to study the distribution of cholesterol in the PM cannot be trusted. Thus, we refer to a review discussing this controversial issue [139]. Based on several reports that, e.g., exosomes contain more than 40 mol% of cholesterol [140], it is difficult to understand that cholesterol can mainly be found in only one of the two leaflets.

### 3.2. Interleaflet Coupling

**Figure 7:**
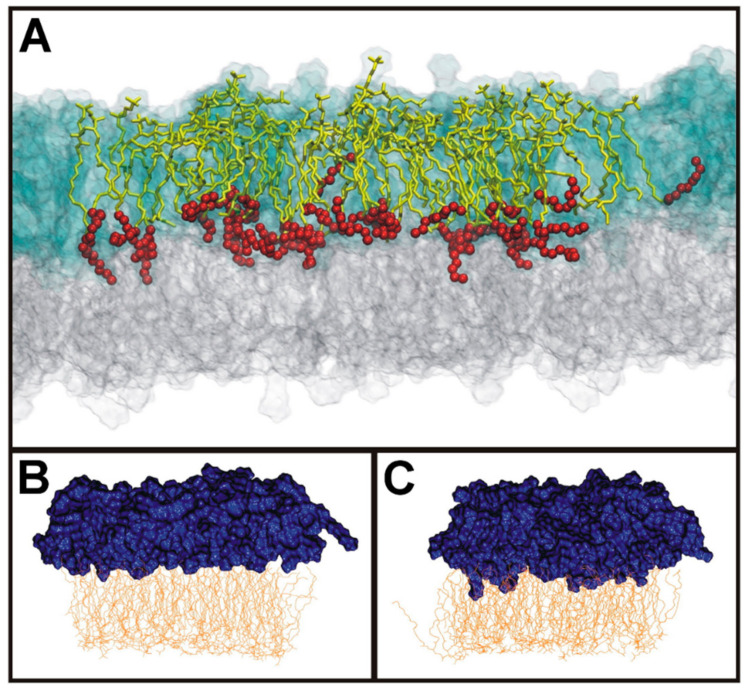
Illustration of interdigitation between the 2 membrane leaflets. (**A**) Multicomponent bilayer where SM d18:1/24:0 are shown as yellow sticks with the 8 last carbon atoms depicted as red balls. Lipids in the outer leaflet are shown as transparent blue glass and lipids in the inner leaflet as transparent grey glass. For clarity, SM d18:1/24:0 are marked only in the central part. (**B**) Model of a bilayer SM d18:1/16:0 and cholesterol in the outer leaflet and with PS 18:0/18:1 and cholesterol in the inner leaflet. (**C**) Similar to (**B**), but SM d18:1/16:0 has been exchanged with SM d18:1/24:0. Blue color is used for the outer leaflet and yellow color is used for the inner leaflet. Note that the N-amidated fatty acyl groups in (**C**) penetrate deeper into the opposite leaflet than in (**B**). For more details, see the open-access article [141] from where this figure is reproduced.

Molecular dynamic simulation studies have proven to be very valuable to study membrane structure, including estimating the degree of interaction between the two membrane leaflets, often referred to as interleaflet coupling or interdigitation. Although such interleaflet coupling is also observed in symmetric bilayer models, stronger coupling was obtained when using asymmetric models [141]. The largest interdigitation was observed when including very long-chain sphingolipids, such as those with 24 carbon atoms, as these chains can cross the membrane midplane and proceed considerably into the opposing leaflet, as illustrated in Figure 7 [141,142,143,144]. As mentioned above, the glycosphingolipids show a bimodal distribution with most of the N-amidated fatty acyl groups containing 16 or 24 carbon atoms. We recently speculated [82] that the glycosphingolipid species have developed this way to obtain a different strength of interleaflet coupling or signaling over the PM.

During recent years, it has become possible to make synthetic lipid vesicles (liposomes) with an asymmetric lipid bilayer. We believe that the use of such liposomes and molecular simulations studies of asymmetric membranes will contribute to important new knowledge about cellular membranes during the next few years. It is important, as discussed in a recent review [82], that such studies are performed using lipid species that are common in cells and with a lipid distribution among the two leaflets that mimic that observed in biological membranes. So far, most studies have been performed using symmetric bilayers made up of lipid species that are not common in cells. Although PLs containing fatty acyl groups such as C16:0, C18:0, C16:1, or C18:1 are present in very low amounts in cells, these species are often the main lipid components in liposomes used to study interactions with various proteins. One should also keep in mind that making standard symmetric liposomes with, e.g., an “endosome-like” or “exosome-like” lipid composition, results in membranes with a composition in the outer leaflet very different from the vesicles these liposomes are intended to mimic.

### 3.3. Methods Used for Quantification of Gb3

Quantification or estimation of the amount of cellular Gb3 have been performed employing many different analytical systems, and such studies have revealed major differences between the total amount of Gb3 and the relative amount of Gb3 species in various cell lines [6]. These methods include the use of MS analyses following lipid extraction of cell lysates. Fluorescently labelled proteins (Shiga toxin, Shiga B, or antibodies) can be used for quantification directly on cells or solid phases, including after the separation of lipids with thin-layer chromatography (TLC). Since Gb3 normally contains mostly N-amidated C16 and C22-24 fatty acyl groups, Gb3 species are separated into two bands on TLC. These can also be quantified with orcinol staining of the carbohydrate moieties or using MALDI-TOF MS directly on the plates. We refer to our earlier review, which features a thorough discussion of the variation of total Gb3 and species content of several cell lines and the methods used for such analyses [6]. We conclude that MS analysis of extracts is the most reliable method for quantification of Gb3 in cells.

### 3.4. Binding Sites for Gb3 on Shiga Toxin

**Figure 8:**
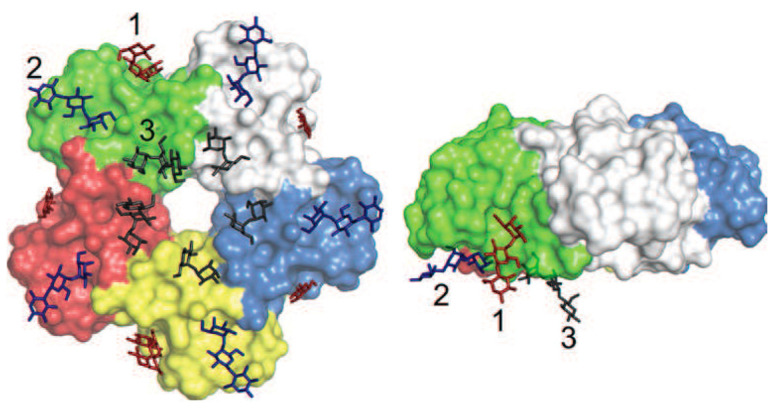
Binding sites for Gb3 to the B-pentamer of Shiga toxin shown by co-crystallization with a Gb3 analog (PDB protein bank IBOS) [147]. Each of the 5 B-subunits of Shiga toxin has the potential to bind 3 Gb3 molecules. Site 1 and 3 bind to the carbohydrates almost perpendicular to the cell surface, whereas site 2 binds to carbohydrates almost parallel to the membrane surface. Reprinted with permission from ref. [148] Copyright 2015 Elsevier.

The binding of Shiga toxin to the glycosphingolipid Gb3 has been studied with a variety of test systems, including cells, liposomes, and solid phase, as discussed thoroughly in an earlier review [6]. Each of the five B-chains have three binding sites for Gb3 resulting in a total of 15 theoretical binding sites at Shiga toxin for Gb3. Two of the three binding sites at each B-chain bind to Gb3 when the carbohydrate structure is oriented close to perpendicular to the membrane surface, whereas one site binds to the carbohydrate part when it is oriented nearly parallel with the membrane surface (Figure 8). This is very interesting since, in the absence of cholesterol, glycosphingolipids have the carbohydrate structure sticking out almost perpendicular to the membrane surface, whereas the presence of cholesterol close to the glycosphingolipid makes the carbohydrate moiety bend and become almost parallel to the membrane surface [145,146]. It is not known how many of these sites, or which sites, that Shiga toxin must bind to in order to be endocytosed. It is also not known how various Gb3 species contribute to binding and uptake of Shiga toxin; we have discussed this in detail earlier [6]. As mentioned above, the dominating Gb3 species in cells are those with N-amidated C16:0, C24:0 and C24:1. Whereas the C16:0 species are expected to penetrate approximately to the midplane of the membrane, the C24:0 and C24:1 species should be able to reach almost halfway into the opposing leaflet. Although the amounts of Gb3 species vary between cell lines and may include minor amounts of other species than the three mentioned, we find it remarkable that C24:1 is the only species containing a double bond and present in high amounts. In the HEp-2 cells we used for most of our studies, the C24:1 species constitutes approximately 50% of the total Gb3 [8]. The importance of the various Gb3 species to the binding and uptake of Shiga toxin, as well as why cells have so much of the N-amidated C24:1 species where the double bond is found almost exactly in the midplane of the membrane (C24:1 is nervonic acid where the double bond is between C15 and C16), are issues which need further investigation.

### 3.5. Lipid Rafts

Lipid rafts are usually described to be enriched in sphingomyelin, cholesterol, and PLs with saturated fatty acyl groups, resulting in most models for lipid rafts containing only PLs with two saturated fatty acyl groups in the inner leaflet. We challenge such a view because many cells have been described to contain a large amount of lipid rafts. At the same time, quantitative lipidomic studies performed during recent years have demonstrated that cells contain very few PLs with two saturated fatty acyl groups [82]. The fact that areas with a large fraction of sphingolipids and cholesterol are less fluid than other parts of cellular membranes may partly be because sphingolipids often contain very few N-amidated fatty acyl groups with double bonds and that PLs with polyunsaturated fatty acyl groups mainly are found in other membrane areas. We also wonder if the view that lipid rafts contain a large fraction of PLs with two saturated fatty acyl groups comes from an early study with detailed quantitative lipidomic analyses of detergent resistant membranes (DRMs). In that study, the DRMs were reported to be highly enriched in saturated PLs, but readers might have overlooked that the authors defined that “a phospholipid was deemed saturated if containing no more than one double bond” [149].

Gb3 is reported to be present in both DRMs and non-DRM domains of several cell lines [150]. There has been much discussion about DRMs as a tool to study lipid rafts [151], but the lipid composition of DRMs may, at least to some extent, reflect the composition of such rafts. Various studies, including some using DRMs, have indicated that Gb3 species present in high lipid order membrane fractions are involved in retrograde transport and toxicity of Shiga toxin [152,153]. Nanodomains containing cholesterol have been reported to be important for Shiga toxin-induced intracellular signaling since the addition of the cholesterol binding substance filipin had an inhibitory effect [77], and extraction of cholesterol using methyl-β-cyclodextrin reduced transport of Shiga toxin to the Golgi apparatus [154].

### 3.6. Signaling into Cells due to Binding of Shiga Toxin to Gb3 in the outer PM Leaflet

The binding of the multivalent AB5 toxins to glycosphingolipid receptors (Shiga toxin binding to Gb3 and cholera toxin binding to GM1) on cells results in intracellular signaling [77,79,155,156,157,158], as discussed in Section 2.1, which raises the question of how binding of toxins to the extracellular leaflet of the PM could result in intracellular signaling. In addition, the binding of antibodies or lectins to these glycosphingolipids results in intracellular signaling [41,159,160], indicating that such signaling effects are due to clustering of the glycosphingolipids. Cholera toxin may trigger intracellular signaling via multiple pathways as it is able to bind also to surface glycoproteins [161,162,163], whereas no other receptors than Gb3 are known for Shiga toxin. We recently discussed the possibility that intracellular signaling following binding of Shiga toxin to Gb3 could be due to clustering of Gb3 in the outer leaflet, which, in turn, due to interdigitation effects, leads to clustering of lipids in the inner leaflet (e.g., PS or PIPs). This inner leaflet clustering of lipids might result in intracellular signaling. Due to the high levels of PS in the inner leaflet, the high amount of PS 18:0/18:1 and the strong interdigitation between this PS species and the very-long chain sphingolipids (Figure 7), we speculated that such clustering of PS could result in binding and activation of some of the many PS binding proteins known to be involved in intracellular signaling (see [82] for a thorough discussion).

### 3.7. Effect on Endocytosis and/or Retrograde Transport due to Manipulations of the Lipidome

Early reports of the effect of Shiga toxin in various cell lines and the content of Gb3 species in these cells have been discussed earlier [6]. In this section, we focus on studies where endocytosis and intracellular transport of Shiga toxin and ricin have been measured after manipulation of the cellular lipidome and the lipid changes have been quantified using MS analyses. An overview of these studies is found in Table 1.

#### 3.7.1. Consequences of Decreasing the Amount of GSLs

To our knowledge, the first study where measurements of endocytosis and intracellular transport were combined with quantitative lipidomics was performed in HEp-2 cells using two inhibitors of sphingolipid synthesis. Fumonisin B1 (10 µM, inhibitor of Cer synthase) and PDMP (1 µM, inhibitor of GlcCer synthase) both reduced the Gb3 level by approximately 50% after 24 h incubation and resulted in a major decrease of Shiga toxin binding/uptake. A further decrease in transport from endosomes to the Golgi apparatus resulted in a large protection against this toxin [164], as summarized in Table 1. There was no change in ricin toxicity in accordance with the reports that retrograde transport of ricin proceeds equally well or is slightly increased in cells lacking glycosphingolipids [165,166].

#### 3.7.2. Studies Related to Ether Lipids

Although PLs with ether-linked alkyl or alkenyl chains (PLs containing an alkenyl group are often called plasmalogens) make up 5–20% of lipids in many cells, there are so far surprisingly few studies of their importance for intracellular transport. The ether-linked chains are most commonly found in the *sn-1* position, whereas the sn-2 position very often contains polyunsaturated fatty acyl (PUFA) chains such as arachidonic acid (C20:4). Ether-linked chains are found in several PL classes, but most often in the PE and PC classes. In most studies, PE ethers have been reported to be the dominant ether lipids, with alkenyl ethers dominating over alkyl ethers [167,168,169,170,171,172]. For reviews of the biological function of ether lipids, see [168,169,173,174,175].

Addition of the ether lipid precursor hexadecylglycerol (HG) to HEp-2 cells resulted in a major increase in the ether lipids and a decrease in the GSLs, with approximately 25% reduction in total Gb3 (no changes in the Gb3 species distribution) [176]. This treatment provided very strong protection of the cells against Shiga toxin and even stronger protection against Stx2 [177].

The reduction in Gb3 obtained with this treatment is too small to explain the very strong protection observed against these toxins, and most of this effect was due to a reduced transport from Golgi to the ER (Table 1). Thus, the increase in ether lipids might provide a protective effect against Shiga toxicity, although further studies are needed to understand the mechanisms behind this effect. In addition, the large increase in LPI obtained as a result of the HG treatment [176] might contribute to this protection (see discussion about addition of lysolipids to cells in Section 3.7.5).

We found it remarkable that an inhibition of GSL synthesis by Fumonisin B1, but not with PDMP, resulted in increased levels of ether lipids [164], and that increasing ether lipids by adding the ether lipid precursor HG also resulted in a decrease of GSLs [176]. Addition of HG to PC-3 cells (which do not contain measurable amounts of Gb3) also resulted in an increase of ether lipids and a decrease in GSLs [170]. Moreover, this treatment of PC-3 cells resulted in an increase of cholesterol, indicating that cholesterol is important for interactions with ether lipids, which has been similarly described for interactions between cholesterol and sphingomyelin [131]. Importantly, these three studies all showed an inverse correlation between the levels of ether lipids and glycosphingolipids. In the next Section 3.7.3, changes in the retrograde transport and lipidome of HEp-2 cells with increased cell density are discussed. Interestingly, this study also showed a small increase in GSLs and a simultaneous small decrease in ether lipids [33], further supporting this apparent co-regulation of ether lipids and sphingolipids. The reason for such a co-regulation is unknown, but it should be noted that the changes in the cell density study were observed without any treatment of cells, and the cells were only grown for an extra day or two. In summary, the studies discussed in this paragraph demonstrate that investigation of Shiga toxin transport has revealed a co-regulation between the levels of sphingolipids and ether lipids.

Recently, Howard Riezman and colleagues reported a similar correlation between ether lipids and sphingolipids in four cell lines in an extensive study. They used a CRISPRi library to repress the expression of 16,000 genes, combined with analyses of sphingolipid-depleted cells (they used myriocin, an inhibitor of the first enzyme in the synthesis of the sphingolipids). They reported a slight but significant increase in PC ether lipids, thus providing another line of evidence for a co-regulation between ether lipids and sphingolipids [184]. It should also be noted that there was no obvious correlation between the amplitude of the decrease in sphingolipids and increase in ether lipids in the four cell lines studied. In addition, alkylhydroxyacetonephosphate synthase (AGPS), a key enzyme in synthesis of ether lipids, was found to be important in promoting the survival of sphingolipid-depleted cells [184]. These authors also showed that ELOV5, an enzyme which is mainly involved in elongation of PUFAs, contributed to making their cells more sensitive to sphingolipid depletion, whereas ELOV6, which mainly acts on elongation of C16-C18 fatty acids, rendered cells more resistant to sphingolipid depletion. These results are in agreement with the changes observed in the cell density study discussed in the next section, where less PUFA-containing and more C18-containing ethers were found at increased cell density [33]. In summary, several studies from two groups have demonstrated an inverse relationship of ether lipids and sphingolipids [33,164,170,176,184]. Furthermore, such changes do not only affect the total amount of ether lipids, but also the species composition. There are, however, major differences between the results reported from the two groups when it comes to the amount of the various ether lipids, and more studies are needed to understand these differences.

Regarding discussions about ether-linked PLs, it is interesting that they behave differently from their corresponding ester-linked analogs regarding how the hydrophobic chains enter membranes. It is now 30 years since magnetic resonance spectroscopy analyses comparing PC with two acyl chains and PE alkenyl ethers indicated that these hydrophobic chains enter membranes differently. The ether chains seem to enter perpendicularly into the membrane, whereas in the PLs with two acyl groups, the first two carbon atoms in the *sn-1* position are almost parallel to the plasma membrane, while the rest of the acyl chain bends into the membrane [185]. Later, atomistic molecular dynamic simulation studies were used to confirm a similar behavior of PE alkenyl ethers and that the presence of these ether lipids resulted in a more densely packed and thicker bilayer than PE with two fatty acyl chains [186]. Thus, the ether lipids show several similarities with the sphingolipids concerning how the hydrophobic chains enter the cell membrane, interact with cholesterol, and contribute to the thickness of the lipid bilayer. For future studies of ether lipids, it is important to be aware of possible differences between alkyl and alkenyl ethers [33,167,184]. It should also be noted that ether lipids are required for generation of glycosylphosphatidylinositol (GPI)-anchored proteins, which can be present in lipid rafts [187].

#### 3.7.3. Effect of Cell Density

Several studies have shown that changes of the cell density in culture could modulate the effect of plant and bacterial toxins, as less toxicity was observed at increased cell density [30,188]. To look more closely into this effect, HEp-2 cells were grown to three different cell densities, from 20–30% density on Day 1 to 80–90% density on Day 3 [33]. As expected, a decrease in Shiga toxin induced cell toxicity was observed with increasing cell density. Increasing cell density also resulted in an increase in Gb3 (no change in the relative amount of Gb3 species) and cholesterol and a decrease in ether lipids, as discussed in Section 3.7.2. Remarkably, the cells with a small increase in Gb3 levels showed some decrease in binding/uptake of Shiga toxin, whereas a stronger reduction of the toxicity was found to be due to a reduced transport from endosomes to Golgi or further to the ER (Table 1). A detailed quantitative lipidomic study showed some changes in a few PA and DAG species which might be related to the decreased Shiga toxicity [33], but further studies are needed to understand why Shiga toxin is much less toxic to cells at a higher cell density.

The group of Lucas Pelkmans later reported an extensive study on cell crowding effects of mouse embryonic fibroblasts either expressing or lacking focal adhesion kinase (FAK) [34]. They reported that more than 1000 genes adapted their transcript abundance to cellular crowding, of which 80% required the presence of FAK to adapt. Notably, most changes reported in the lipidome of the fibroblasts as a function of increased cell density [34] were different from those reported in the study with HEp-2 cells [33]. Thus, increasing the cell density of various cell lines has been reported to result in different changes of the lipidome, and more studies are needed to understand how and why such changes occur.

#### 3.7.4. Glucose Analogues Induce Protection against Shiga Toxins

For more than 50 years, 2-deoxy-glucose (2-DG) has been used as an inhibitor of glycolysis. During the last 15 years, 2-DG has been shown to affect several other cellular mechanisms, such as autophagy, apoptosis, and cell cycle control, and has also been shown to suppress the expression of Gb3 synthase (references in [178]). This led us to study if it had any effect on Shiga toxicity in HEp-2 cells. Although 2-DG had only minor effects on endocytosis and transport to the ER in the HEp-2 cells, it provided strong protection against Shiga toxicity, and this effect was shown to be mainly due to an inhibition of the release of Shiga toxin A1 in ER (Table 1) [178]. A few percent of 2-DG was incorporated into GSLs, but the treatment with 2-DG had only minor effects on other lipid classes.

2-Fluoro-2-deoxy-glucose (FDG) is another glucose analogue that is very similar to 2-DG (one H exchanged with F), and [^18^F]FDG is the most common imaging agent used for positron emission tomography (PET) for multiple cancers [189,190]. Remarkably, FDG was found to protect HEp-2 cells much more strongly than 2-DG, as 1 mM FDG provided protection similar to that obtained by 10 mM 2-DG. Furthermore, the protection with FDG was shown to be mainly due to inhibition of GlcCer synthase [179]. It should be noted that these two glucose analogues were shown to protect also several other cell lines against Shiga toxin, and that a very similar protection was observed for Shiga toxin and Stx2, whereas they did not have any protective effect against ricin [178,179].

#### 3.7.5. Substances Affecting the Membrane Fluidity

Addition of several lysolipids to HEp-2 cells resulted in a decreased cellular binding of Shiga toxin, Stx2, and anti-Gb3 IgM [180]. The effect increased with the increasing size of the head groups, i.e., LPI > LPC > LPS > LPE >> LPA, with no effect observed for LPA (Table 1). The effect was also dependent on the fatty acyl groups. A stronger effect was obtained with LPC 18:0 than with LPC 18:1, further evidencing the importance of the conical structure for this effect. Most studies were performed using LPI 18:0. The experiments showed that prebound Shiga toxin was released following addition of the lysolipid, and that the LPI effect was observed both in fixed cells or after depletion of ATP, demonstrating that the effect was independent of signaling or membrane turnover. LPI addition resulted in the cell surface rounding up, with less philopodia. Furthermore, studies with the fluorescent probe NR12S, which is predicted to stay in the outer leaflet, revealed that LPI induced lipid disorder in the PM. There was no change in lipid disorder after addition of LPE. LPI and methyl-β-cyclodextrin both increased the lipid disorder, but led to opposing effects on toxin binding, showing that the reduction in lipid order did not seem to be important for the binding. Addition of methyl-β-cyclodextrin did not affect binding of Shiga toxin but reversed the effect of LPI, suggesting that LPI altered Gb3 receptor conformation and/or distribution. The observed decrease in lipid packing might facilitate interactions of Gb3 with cholesterol, binding the carbohydrate and thereby inhibiting the Shiga toxin binding, an effect which was reversed by addition of methyl-β-cyclodextrin. Addition of LPI did not have any effect on binding of ricin [180].

In a follow-up study, lysolipids with large head groups were shown to perturb clathrin-mediated endocytosis. The effect depends on the cells used. The largest effects were observed for HEp-2, HeLa, and SUM-159 cells, and smaller effects were observed for SK-BR3, U-2 OS, and PC-3 cells. Less force (50% reduction) was needed to pull tubules outward from HEp-2 cells following the addition of LPI, whereas no reduction in this force was observed following the addition of LPE [181]. Moreover, the addition of LPI resulted in increased lifetimes for AP-2 pits, and the effect on ricin uptake was much smaller than for transferrin, indicating that the lysolipid effect was larger for clathrin-dependent than for clathrin-independent endocytosis. In this study, the effect was also lower when using lysolipids with smaller head groups (LPE and LPA) or unsaturated lipids (LPC 18:1). As expected, there was a stronger effect of Latrunculin B on transferrin endocytosis after LPI addition, which agrees with data showing that actin requirement for endocytosis is increased at high membrane tension [191].

In the studies with lysolipids and toxins described above, these lipids did not only seem to affect binding but also had an additional effect on retrograde transport and toxicity. A study in yeast showed that lysolipids facilitated COPII vesicle formation and anterograde transport from ER to the Golgi [192]. An effect of lysolipids on anterograde and retrograde transport may be related. In both studies, it was speculated that the conical structure of the lysolipids is important for these effects, but further studies are needed to describe the precise mechanism.

After preincubation with HeLa cells, the antitumor drug 2-hydroxyoleic acid (OHOA) was shown to potently increase retrograde transport of ricin from endosomes to Golgi and further to the ER and result in a major increase in sensitivity to this toxin, whereas Shiga toxin transport to the Golgi was slightly reduced as evaluated with sulfation experiments [171]. After incubation of the cells with 12.5 µM OHOA for 3 h, results showed that 6% of the cellular lipids contained this fatty acyl group. Furthermore, a reduced PM lipid packing was observed similar to that described above following the addition of lysolipids. The differential effect observed with OHOA on transport of ricin and Shiga toxin is in line with data obtained in an earlier study on the treatment of HeLa cells with polyunsaturated fatty acids [182]. Remarkably, there was no effect on toxin transport by oleic acid. It is not known how the OH group of OHOA could contribute to such large effects as those observed following addition of OHOA to cells. OHOA did not promote the recruitment of SNX1 or SNX2 to the endosomes (SNXs have been shown to be involved in ricin transport from endosomes to Golgi [101]), but an increased endosomal localization of the retromer component VPS35 was observed. Thus, it is possible that OHOA promotes ricin transport by increasing membrane affinity for proteins mediating retrograde transport.

Addition of polyunsaturated fatty acids (PUFAs, i.e., C20:5 and C22:6) to HeLa cells resulted in a 10-fold protection against Shiga toxin [182]. Both the internalization of this toxin and endosome-to-Golgi transport were reduced by PUFAs, and these reductions could together explain the reduced toxicity (Table 1). Also, cholera toxin internalization was reduced by PUFA treatment. Ricin cytotoxicity was not affected, demonstrating that PUFAs do not cause a general block in retrograde transport to the endoplasmic reticulum.

### 3.8. Modifications of the Lipidome: Changes as Expected?

Although interfering with lipid metabolism using inhibitors, siRNA, or by supplying cells with some lipids may provide important information about the role of lipids for endocytosis and intracellular transport, we would also like to warn that the effects observed following such treatments might be due to other changes in the lipidome than those expected. Thus, careful analyses should be carried out to check if the changes in the lipidome are as expected. An excellent example of this is a study by the group of Clifford Lingwood [193]. They used statins, known inhibitors of the rate determining enzyme in cholesterol synthesis, and observed that the retrograde transport of Shiga toxin (and cholera toxin) to Golgi and ER was blocked. By looking carefully into what happens with these cells, they observed that the statin treatment resulted in a partial relocation of the GlcCer synthase, elevated levels of this enzyme, and a several-fold increased level of GlcCer in all nine cell lines studied. There were also some changes in the total level of several other GSL classes but no changes in the relative amounts of the species of each class. The total cellular cholesterol level was unaffected by the statin treatment, which is in agreement with earlier reports that inhibition of cholesterol synthesis triggers an increased expression of the LDL receptor to maintain the cellular cholesterol level [194], while the cholesterol pool in the trans-Golgi network was dissipated. They showed that the changes in GSL and GlcCer synthase were independent of cholesterol synthesis inhibition, but that these effects surprisingly resulted from an aberrant Rab GTPase prenylation. Thus, they revealed an unexpected link between Rab prenylation (geranylgeranyl is an intermediate in the cholesterol biosynthesis) and regulation of GSLs and retrograde trafficking.

We recently published that inhibitors of diacylglycerol kinase (DAG kinase) and phospholipase D (PLD) strongly increased the retrograde transport of ricin by affecting the endosomal sorting toward the Golgi apparatus [183]. Treatment with these inhibitors strongly affected the endosome morphology by increasing endosomal tubulation and size. Quantitative lipidomic studies of the inhibitor treated cells showed a lipidome as expected when using the DAG kinase inhibitor (increased DAG), whereas the PLD inhibitor resulted in a lipidome very different from that expected. There was a small temporary increase in PA, but it then decreased to control levels after 3 h. Unexpectedly, there was an increase in DAG and phosphatidylglycerol (PG), and the species which were increased, clearly indicated that this was due to the metabolism of PC species. We speculate that the use of the PLD inhibitor resulted in compensatory mechanisms where the cells aimed at keeping the cellular PA levels relatively constant. Clearly, these lipid data demonstrate the need to perform quantitative lipidomic analyses before conclusions are drawn from such studies. Furthermore, siRNA was used to knock down different isoenzymes of DAG kinase and PLD. In addition to demonstrating that several of these isoenzymes were involved in regulating ricin transport to the Golgi, the experiments revealed one case where knock-down of one isoenzyme of DAG kinase resulted in increased levels of four other isoenzymes [183], thus demonstrating compensatory mechanisms in such studies.

### 3.9. The Need for Future Studies and a Focus on the Importance of Lipid Species

Based on the discussion above, there is no doubt that we have much to learn about the importance of membrane lipids for endocytosis and intracellular membrane transport. Although the development of improved MS analyses during recent years has opened new opportunities to perform quantitative analyses of hundreds of lipid species in one sample, we are still in a very early process of learning about the importance of lipid species in cell biology. Although much has been learned about binding of cholesterol to different proteins [195,196,197], binding of other lipids to various lipid transfer proteins [131], and binding of proteins to lipid classes such as PIPs, PS, and PA [82,198,199], very little is known about interactions between specific lipid species and proteins. To our knowledge, the first demonstration of the importance of one lipid species in biology was the report that PG 16:0/18:1 was an essential lipid close to the O_2_-binding site of cytochrome c oxidase. Remarkably, the C18:1 was not the very common C18:1 oleate (cis Δ^9^), but the uncommon C18:1 vaccinate (cis-Δ^11^), showing the importance of one double bond being placed two carbon atoms further away from the PG head group [200]. Later, it was shown by Brita Brügger and colleagues that one SM species (SM d18:1/18:0) was essential for binding to the transmembrane domain of the COPI machinery protein p24. They suggested a role for this SM species in regulating the equilibrium between the inactive monomeric and an active oligomeric form of this protein in COPI-dependent transport from Golgi to the ER [201]. So far, to our knowledge, there is no other example clearly demonstrating the effect of specific lipid species, but we recently summarized several interesting properties regarding PS 18:0/18:1 and speculated about the contribution of this species to endocytosis and intracellular transport, including its possible role in endocytosis of Shiga toxin [82].

## 4. Conclusions

Our understanding of how protein toxins act on cells has undergone a remarkable development during the last four decades. The present review discusses several examples of how Shiga toxin and ricin have contributed to such knowledge, as well as to our understanding of endocytosis and intracellular transport in general. During the last 10–15 years, there has been a remarkable improvement in MS analyses and the possibilities to benefit from such analyses to learn more about cellular mechanisms. It is important to stress that we are still in a very early process of using of quantitative lipidomic studies related to cellular functions. In our opinion, it would be very surprising if many more examples of the biological roles of specific lipid species were not discovered during the next years. Each cell synthesizes several hundred or thousand lipid species. Why should cells use energy to synthesize so many lipids if not many of them are needed for specific purposes? Thus, we foresee major advances in new knowledge regarding the importance of lipid species for membrane transport during the next years. To this end, it is important that the methods used for sample preparation and MS analyses become better validated and documented than often seen today. It is imperative that scientists can trust that the data are reproducible. It is also important that such quantitative data are made available for everybody, as it may take several years before we are able to fully interpret such data. Based on our experience, it is not sufficient to have modern MS equipment but it is also necessary to have solid knowledge about which lipids one can expect to find in biological samples. We also expect molecular simulation studies and the use of synthetic models to be important for obtaining new knowledge in this field. We stress the importance of basing such models on asymmetric membranes composed of lipid species commonly available in cells.

## Figures and Tables

**Table 1:** Summary of studies aiming to reveal correlations between endocytosis, intracellular transport, and cellular lipids. Upward arrows mark increased level of lipids, binding, or steps leading to toxicity, and downward arrows mark the opposite. The number of arrows indicate the size of the effects. Empty boxes mean not measured, and the sign for similar (~) means no or very minor changes. “Endo **→** Golgi” means transport from endosomes to the Golgi apparatus. “Golgi **→** ER” means transport from the Golgi apparatus to the endoplasmic reticulum.

Treatment	Binding	Uptake ^1^	Endo → Golgi	Golgi → ER	Toxicity	Cer	GlcCer LacCer	Gb3	Acyl PL	Ether PL	Other Information
Fumonisin ^2^	Stx ↓↓	~	Stx ↓↓↓		Stx ↓↓↓	↓↓	↓↓	↓↓	PE ↑↑	PE ↑↑ PC ↓	No effect on ricin
PDMP ^3^	Stx ↓↓	~	Stx ↓↓↓		Stx ↓↓	~	↓↓	↓↓	~	~	No effect on ricin
HG ^4^	Stx ↓	~	~	Stx ↓↓	Stx ↓↓↓	~ (↑)	↓↓	↓↓	PI ↑↑ LPI ↑↑↑	PE ↑↑ PC ↑↑	No effect on ricin. See also ^4^
Cell density ^5^	Stx ↓↓	~	~	~	Stx ↓↓	↑	↑	↑	PA↑↑ PI + PE ↓	PE ↓ PC ↓	No effect on diphtheria toxin
2-DG ^6^	~	~	~	~	Stx ↓(↓)	~ (↑)	Cont. 3% 2-DG	↓ 1% 2-DG			Inhibits release of Shiga toxin A1 in ER
FDG ^7^	Stx ↓	~	~	Stx ↓	Stx ↓↓↓	~	↓↓↓	↓↓↓	PI ↑		Inhibits GlcCer synth.
Lyso PL ^8^ (LPI 18:0)	Stx ↓↓		Stx ↓↓↓		Stx ↓↓↓						PM lipid packing ↓
Polyunsaturated FA ^9^	Stx ↓	Stx ↓	Stx ↓		Stx ↓↓↓						Varying effect on other toxins (see ^9^)
OHOA ^10^	~	~	Ricin ↑↑	~	Ricin ↑↑	~	~		~ (see ^11^)	~ (see ^11^)	PM lipid packing ↓
DAG kinase and PLD ^11^		~	Ricin ↑↑	~	Ricin ↑	~	~		~ (most)	~	See text for DAG, PA and PG

^1^ In this column, the similar sign (~) means uptake changed similar to binding ^2^ Fumonisin B1: 10 µM, 48 h, HEp-2 cells [164]. ^3^ DL-threo-1-phenyl-2-decanoylamino-3morpholino-1-propanol: 1 µM, 24 h, HEp-2 cells [164]. ^4^ *sn-1-*O-hexadecylglycerol: 20 µM, 24 h, HEp-2 cells. No or only minor effect on cytotoxicity by ricin, cholera toxin, or diphtheria toxin. No effect on transferrin endocytosis. Toxicity also shown for Stx2 in HEp-2, HBMEC and HBMEC-2 cells [176,177]. ^5^ Data in table shown for HEp-2 cells grown for 1, 2, or 3 days to obtain a cell confluency of 20–30% on Day 1 and 80–90% on Day 3. Data given for changes due to increased cell density. Similar toxicity data were shown in HeLa cells. TLC analyses revealed less Gb3 at high density in HeLa cells and close to similar amounts in HEp-2 cells [33]. ^6^ 2-Deoxy-D-glucose: 10 mM, 4 h and 24 h, HEp-2 cells. Several changes in the lipidome; 1–3% of GSLs contain 2-DG. Similar toxicity observed with Stx2 and diphtheria toxin, but no change in toxicity with ricin. 2-DG also protected HT-20 and SW480 cells against Shiga toxicity. 2-DG decreased transferrin endocytosis, but less than that of Shiga toxin [178]. ^7^ 2-Fluoro-2-deoxy-D-glucose: 1 mM, 24 h, HEp-2 cells. FDG inhibits GlcCer synthase; effect on GSLs observed after 24 h, not after 4 h. Protection against Stx2 similar to protection against Shiga toxin in HEp-2 cells, but only a very weak protection against ricin and no protection against diphtheria toxin. Similar protection against Shiga toxin in MCF-7, HT-29, and HBMEC cells [179]. ^8^ Lyso PL: Data are shown for many different lyso PLs, 5–20 µM, 30 min, Hep-2 cells. Largest effects observed with the most conical lyso PL, i.e., those with the largest head groups (e.g., LPI 18:0 with a large head group: LPI > LPS > LPC >LPE > LPA). Symbols in the table are showing changes with LPI 18:0. The effects were reversed by the addition of methyl-β-cyclodextrin. Similar effects observed with Stx2 [180]. In a follow-up article, these lyso PLs were shown to perturb clathrin-mediated endocytosis, with the largest effects observed with the lipids with the largest head groups [181]. ^9^ Polyunsaturated FA: 50 µM EPA (20:5) or DHA (22:6), 2 days, HEp-2 cells. Similar reduced toxicity observed with cholera toxin, whereas a slightly increased toxicity was observed with ricin. Only minor decrease in transferrin endocytosis [182]. ^10^ 2-Hydroxyoleic acid (Minerval^®^): 12 µM, 3 h, HeLa cells. OHOA incorporated into ~11% of acylated PL and 10% of ether lipids. A similar toxicity was observed in HEp-2 and U2-OS cells [171]. ^11^ DAG kinase and PLD (phospholipase D): Use of inhibitors and siRNA to modify the levels of DAG and PA in HEp-2 cells. Inhibitors led to increased transport to Golgi and increased endosome size and tubulation. Effect increased by combining the inhibitors. No changes in recycling or degradation of ricin [183]. See the main text for further details.

## Data Availability

No new data were created or analyzed in this study. Data sharing is not applicable to this article.
